# Association of Lesion Location and Fatigue Symptoms After Ischemic Stroke: A VLSM Study

**DOI:** 10.3389/fnagi.2022.902604

**Published:** 2022-06-29

**Authors:** Jinjing Wang, Mengmeng Gu, Lulu Xiao, Shiyi Jiang, Dawei Yin, Ye He, Peng Wang, Wen Sun, Xinfeng Liu

**Affiliations:** ^1^Department of Neurology, Affiliated Jinling Hospital, Medical School of Nanjing University, Nanjing, China; ^2^Department of Neurology, Nanjing First Hospital, Nanjing Medical University, Nanjing, China; ^3^Stroke Center & Department of Neurology, The First Affiliated Hospital of USTC, Division of Life Sciences and Medicine, University of Science and Technology of China, Hefei, China; ^4^Department of Radiology, The First Affiliated Hospital of USTC, Division of Life Sciences and Medicine, University of Science and Technology of China, Hefei, China

**Keywords:** lesion location, voxel-based lesion-symptom mapping, poststroke fatigue, acute ischemic stroke, neuroimaging

## Abstract

**Background:**

Poststroke fatigue (PSF) is a common symptom in stroke survivors, yet its anatomical mechanism is unclear. Our study was aimed to identify which brain lesions are related to the PSF in patients with acute stroke.

**Method:**

Patients with first-ever acute ischemic stroke consecutively admitted from the first affiliated hospital of the University of Science and Technology of China (USTC) between January 2017 and June 2020. Fatigue was scored using the Fatigue Severity Scale. All the participants were assessed by 3.0 T brain MRI including diffusion-weighted imaging. The infarct lesions were delineated manually and transformed into a standard template. Voxel-based lesion-symptom mapping (VLSM) was applied to investigate the association between lesion location and the occurrence and severity of fatigue. The same analyses were carried out by flipping the left-sided lesions. Multivariate logistic regressions were applied to verify the associations.

**Results:**

Of the 361 patients with acute stroke, 142 (39.3%) patients were diagnosed with fatigue in the acute phase and 116 (35.8%) at 6 months after the index stroke. VLSM analysis indicated clusters in the right thalamus which was significantly associated with the occurrence and severity of PSF at 6-month follow-up. In contrast, no significant cluster was found in the acute phase of stroke. The flipped analysis did not alter the results. Multivariate logistic regression verified that lesion load in the right thalamus (OR 2.67, 95% CI 1.46–4.88) was an independent predictor of 6-month PSF.

**Conclusion:**

Our findings indicated that lesions in the right thalamus increased the risk of fatigue symptoms 6 months poststroke.

## Introduction

Poststroke fatigue (PSF) was described as a subjective feeling of exhaustion after stroke, which could not be ameliorated by rest (Kutlubaev and Mead, 2012). It has a detrimental impact on functional recovery, quality of life, and long-term outcomes of stroke survivors (Maaijwee et al., 2015; Oyake et al., 2021; Rutkowski et al., 2021). Some observational studies indicated that the occurrence of PSF involved the biological (Kutlubaev et al., 2012; Gu et al., 2021), physiological (Wu et al., 2014), and psychological factors (Suh et al., 2014; Tao et al., 2021). However, the underlying mechanism of PSF remains unclear (Chen et al., 2015). A deeper understanding of the topological mechanism of PSF might provide therapeutic targets for developing effective strategies.

The relationship between lesion location and PSF remains controversial (De Doncker et al., 2018). Brainstem lesions, thalamus (Visser et al., 2019), white matter lesions (Tang et al., 2014), corona radiate, and internal capsule (Wei et al., 2016) were reported to be associated with PSF in the previous studies. However, other studies suggest that lesion location is not a determinant of the development of fatigue (Kutlubaev et al., 2012). One of the main factors leading to such discrepancies is that the infarct lesions involved in these studies mainly based on the visual assessment thus lack of anatomical precision.

Voxel-based lesion-symptom mapping (VLSM) is an important method to study the clinical–anatomical correlations in patients with stroke, which can automatically localize the cognitive behavior functions in the human brain (Bates et al., 2003). To elucidate the question whether lesion location is a biological factor for PSF, in our study, we applied the VLSM method to investigate this lesion-behavior associations during the acute phase and at 6-month follow-up after the index stroke.

## Methods

### Study Population

Patients with acute ischemic stroke were admitted from the Department of Neurology, the First Affiliated Hospital of University of Science and Technology of China (USTC) between January 2017 and June 2020. Patients were admitted to the hospital within 14 days after stroke onset and followed-up for 6 months. The inclusion criteria for this study were (1) age ≥18 years; (2) first-ever stroke; (3) acute ischemic stroke confirmed by magnetic resonance imaging (MRI); and (4) ability and willingness to participate in this study. Subjects were excluded because of the inability to perform behavioral testing, presence of cancer or other chronic disease (including heart failure, chronic liver, renal insufficiency, and thyroid dysfunction), pre-existing neurological disorders (including Parkinson's disease, multiple sclerosis, and myasthenic), history of fatigue or other mental disease (including anxiety, depression, drug abuse, and schizophrenia) before the index stroke, had taken antidepressants, and contraindications to MRI (including metal implants, pregnancy, and claustrophobia). Moreover, all the patients were assessed with the Mini-Mental State Examination (MMSE), and those with an MMSE score of ≤ 10 or an MMSE score of 11–23, but considered to have poor-cognitive ability were excluded (Braley et al., 2014).

The study was approved by the local ethics committee and informed consent for study participation was obtained from all the participants or their legally designated surrogates.

### Baseline Assessments

Information on demographic data, previous histories, and vascular risk factors was obtained in an interview process. Body mass index (BMI) was defined as the weight in kilograms divided by the square of the height in meters (Criqui et al., 1982). The stroke subtype was classified using the Trial of Org 10172 in Acute Stroke Treatment (TOAST) criteria (Adams et al., 1993). The stroke severity at admission was assessed according to the National Institutes of Health and Stroke Scale (NIHSS) score.

### Behavioral Testing

The fatigue symptoms were measured using the Chinese version of the Fatigue Severity Scale (FSS), which has been confirmed and validated in the previous study (Wang et al., 2016). The scale consists of nine questions graded on a 1–7 scale with higher values indicating more severe symptoms of fatigue. The FSS score was transformed into a dichotomous variable with a mean ≥ 4, which was considered as an indicative variable of PSF and was also used as a continuous variable to estimate the severity of fatigue in subsequent analysis (Wang et al., 2021). The symptoms of depression, anxiety, and social interpersonal relationships were assessed using the 24-item Hamilton Depression Scale (HAMD-24; Hamilton, 1980), 14-item Hamilton Anxiety Scale (HAMA-14; Maier et al., 1988), and Lubben Social Network Scale (LSNS; Lubben, 1988), respectively.

### Follow-Up

The patients were evaluated once again for FSS and the functional status was quantified with the modified Rankin Scale (mRS) at 6 months after index stroke onset. All the assessments were blinded to baseline characteristics, the data entry and quality control are completed by separate researchers independently of the assessments.

### MRI Acquisition and Preprocessing

All the patients were scanned with 3.0 tesla MRI scanner (Discovery MR 750, GE Healthcare, Milwaukee, WI, USA) within 3 days after admission. The sequences mainly included diffusion-weighted images (DWI), T1-weighted spin echo, and fluid-attenuated inversion recovery (FLAIR).

**Figure 1:**
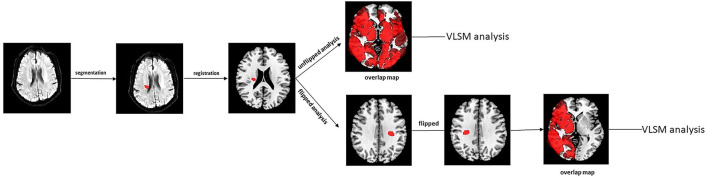
The flow chart of image preprocessing procedures for VLSM analysis. VLSM, voxel-based lesion-symptom mapping.

Infarct segmentation and registration to the standard MNI-152 brain template were performed in accordance with the previous published articles (Biesbroek et al., 2019). First, acute infarct lesions were segmented manually on DWI sequence using the ITK-SNAP software (Solana et al., 2021). Accordingly, a lesion mask was generated for each subject. Next, the segmented maps and recording scans were registered to T1 1-mm MNI-152 brain template with the RegLSM toolbox (Weaver et al., 2019) in SPM12 running under MATLAB R2017a. All the registration results were checked visually by an experienced radiologist blinded to the behavioral data. Then, a summation map of all infarct lesions was created and lesion volume was calculated using the successful registrations. The lesion volume was obtained by counting the number of lesioned voxels and multiplying by each voxel volume (Ye et al., 2017). A flowchart for VLSM analysis was displayed in Figure 1.

### Voxel-Based Lesion-Symptom Mapping

To identify whether lesioned brain voxels were associated with fatigue symptoms, we performed VLSM analysis using the MATLAB package NiiStat (Padmanabhan et al., 2019), controlled for lesion volume. Voxels damaged in which fewer than five patients were excluded from the VLSM analyses (Sperber and Karnath, 2018). We tested for differences between the two groups (PSF+/PSF-) by means of 1-tailed Liebermeister test and the Brunner–Munzel test for continuous behavioral data. The left-sided lesions were flipped to right hemisphere alongside the midsagittal line, and the same VLSM analysis was performed to investigate lesion location and PSF in both in the acute phase and 6-month follow-up after stroke onset. Anatomic labeling was performed with Automated Anatomical Labeling (AAL) atlas, which is included in the NiiStat software. VLSM results were visualized in MRIcron software overlaid onto a standard T1-weighted MR image (ch2 template). All the statistical tests were one-tailed and a *p* < 0.05 was considered statistically significant. Correction for multiple comparisons [family-wise error rate (FWE), *P* < 0.05] was used to threshold the analysis.

### Statistical Analyses

Continuous variables are presented as the means ± SD or medians (interquartile ranges), and categorical variables are presented as numbers (percentages). Categorical variables were compared with the χ^2^ test, and continuous variables were compared with Student's *t*-test or the Mann–Whitney *U*-test, as appropriate.

The participants were divided into two groups (VLSM+/VLSM–) according to the significant lesions. *Post-hoc* analysis was carried out to analyze the clinical characteristics differences between VLSM+ and VLSM– group. This new variable was included into multivariate logistic regression with three models, subsequently. Model 1 adjusted for age and sex; model 2 adjusted for age, sex, and lesion volume; and model 3 adjusted for age, sex, lesion volume, BMI, hypertension, diabetes, hyperlipidemia, smoking, drinking, stroke classification, NIHSS score, HAMD score, and Lubben score. The results of multivariate logistic regression were displayed as forest plot. All the clinical data were analyzed using SPSS Statistic 26.0 software (IBM Corp., Armonk, NY). Two-sided values of *p* < 0.05 were considered statistically significant.

## Results

### Demographics and Clinical Characteristics

According to the aforementioned eligibility criteria, a total of 633 consecutive patients was screened in the acute phase of stroke, 482 eligible patients were enrolled to the study. Of these, 121 patients were excluded due to incomplete segmentation (*n* = 54) or registration errors (*n* = 67). There were 361 patients left for VLSM analysis in the acute phase of stroke. During the 6-month follow-up period, 14 patients had a stroke recurrence, 7 withdrew from the study, 16 patients did not respond because of deterioration of physical function. As a result, the remaining 324 patients complete VLSM analysis at 6-month follow-up. A flowchart of patient selection is provided in Supplementary Figure 1. There were no significant differences in the clinical characteristics between the enrolled and excluded patients (Supplementary Table 1).

The prevalence of PSF was 39.3% in the acute phase following stroke and 35.8% at the 6-month follow-up. The univariate analysis indicated that most of the demographics and clinical characteristics were similarly homogenous between patients with and without PSF, with the exception of the HAMD and HAMA scores in the acute phase of stroke. Significant differences were found in the lesion side, HAMD and HAMA scores between fatigue group and non-fatigue groups at 6-month follow-up (Table 1).

**Table 1:** Baseline characteristics and neuropsychological scores of poststroke fatigue in the acute phase and 6-month follow-up.

	**PSF in the acute phase**		***p*** **-value**	**PSF at 6-month follow-up**		***p*** **-value**
	**PSF– (*n* = 219)**	**PSF+ (*n* = 142)**		**PSF– (*n* = 208)**	**PSF+ (*n* = 116)**	
Age, mean (SD), y	60.4 (12.1)	58.2 (13.2)	0.118	59.6 (12.6)	58.4 (12.7)	0.391
BMI, mean (SD), kg/m^2^	25.1 (3.4)	24.6 (3.2)	0.214	24.9 (3.4)	24.5 (3.2)	0.298
Male, *n* (%)	144 (65.8)	86 (60.6)	0.316	133 (63.9)	72 (62.1)	0.737
Hypertension, *n* (%)	164 (74.9)	96 (67.6)	0.132	154 (74)	75 (64.7)	0.075
Diabetes, *n* (%)	83 (37.9)	60 (42.3)	0.409	77 (37)	41 (35.3)	0.764
Hyperlipidemia, *n* (%)	49 (22.4)	23 (16.2)	0.151	47 (22.6)	19 (16.4)	0.183
Smoking, *n* (%)	77 (35.5)	48 (34.3)	0.817	76 (36.9)	38 (33.3)	0.524
Drinking, *n* (%)	54 (24.9)	33 (23.6)	0.778	51 (24.8)	26 (22.8)	0.696
Time from onset to imaging, median (IQR), day	3 (2–5)	3 (2–5)	0.890	3 (2–5)	3 (2–5)	0.350
Time from onset to behavioral testing, median (IQR), day	13 (12–15)	13 (12–15)	0.725	13 (12–15)	13 (12–15)	0.702
Time from onset to follow up, median (IQR), day	184 (183–185)	184 (182–185)	0.515	184 (182–185)	184 (183-185)	0.372
Lesion volume, median (IQR), ml	2.5 (1.0–6.7)	3.1 (1.0–6.5)	0.587	2.4 (1.0–6.6)	3.9 (1.2-7.2)	0.112
Lesion side, *n* (%)			0.311			0.010
Left	102 (46.6)	58 (40.8)		108 (51.9)	42 (36.2)	
Right	105 (47.9)	79 (55.6)		89 (42.8)	70 (60.3)	
Bilateral	12 (5.5)	5 (3.5)		11 (5.3)	4 (3.4)	
TOAST, *n* (%)			0.750			0.144
LAA	95 (43.4)	67 (47.2)		96 (46.2)	46 (39.7)	
SAD	47 (21.5)	27 (19.0)		45 (21.6)	20 (17.2)	
Others^a^	77 (35.2)	48 (33.8)		67 (32.2)	50 (43.1)	
NIHSS at admission, median (IQR)	3 (1–5.3)	3 (1–7)	0.084	3 (1–6)	3 (1–6)	0.863
mRS at discharge, median (IQR)	1 (1–3)	1 (1–3)	0.602	1 (1–3)	1 (1–3)	0.854
LSNS, median (IQR)	28 (17–38)	28 (17–34)	0.531	28 (18–38)	28.5 (15–34.8)	0.177
HAMA, median (IQR)	4 (1–9.8)	6 (2–15)	0.011	4 (1–7)	7 (2–16)	<0.001
HAMD, median (IQR)	4 (2–8)	5.5 (2–12)	0.013	3 (1.8–7.0)	6 (3–13)	0.001

*BMI, body mass index; HAMA, Hamilton anxiety scale; HAMD, Hamilton depression scale; IQR, interquartile range; LAA, large artery atherosclerosis; LSNS, Lubben social scale; mRS, modified Rankin Scale; NIHSS, NIH Stroke Scale; PSF, poststroke fatigue; SAD, small artery occlusion; SD, standard deviation; TOAST, Trial of Org 10172 in Acute Stroke Treatment*.

a *Others, cardioembolism, stroke of other determined cause, and stroke of undetermined cause*.

### The Results of VLSM Analysis

**Figure 2:**
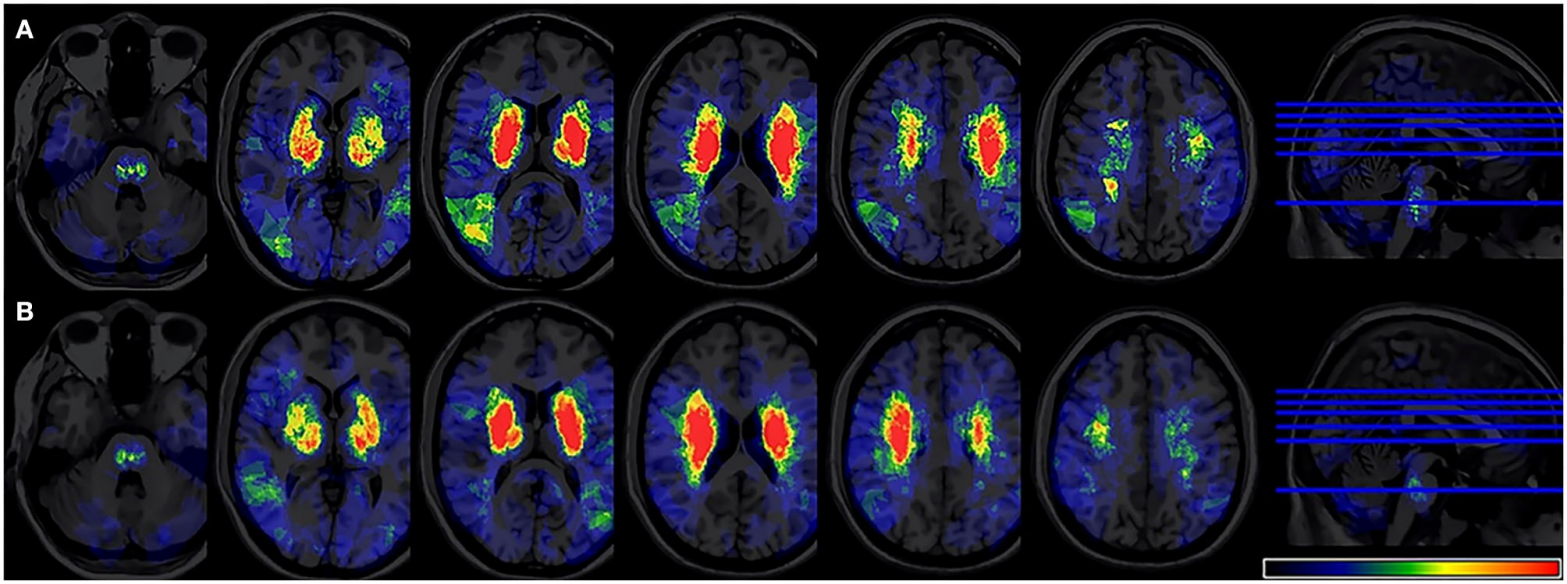
Lesion probability map. **(A)** A total of 361 patients were included in the acute phase VLSM analysis. **(B)** A total of 324 patients included 6-month follow-up VLSM analysis. The color represents the frequency of overlap. VLSM, voxel-based lesion-symptom mapping.

The overlapping lesions in the brains of all the stroke patients included the corona radiata, midbrain, temporal lobes, internal capsule, thalamus, globus pallidus, putamen, insula, occipital lobes, and cerebellum. Overall, infarcts in the basal ganglia areas accounted for a relatively large proportion of the total rate. All the infarct distributions are displayed in Figure 2. The number of overlapping lesions is illustrated by different colors indicating increasing frequency from black to red.

**Figure 3:**
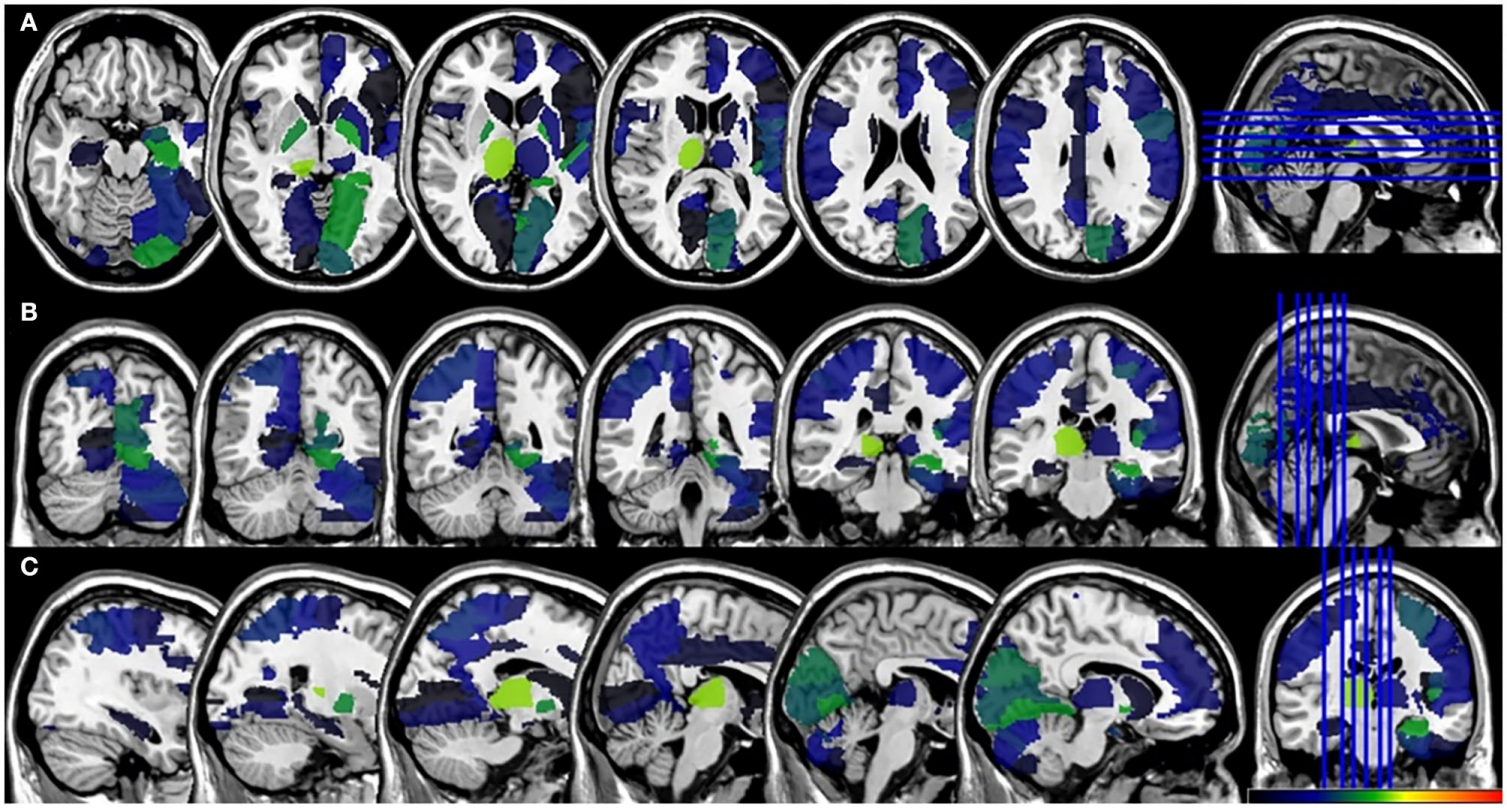
VLSM results for the poststroke fatigue in the acute phase. **(A)** axial plane, **(B)** coronal plane, and **(C)** sagital plane. VLSM, voxel-based lesion-symptom mapping.

**Figure 4:**
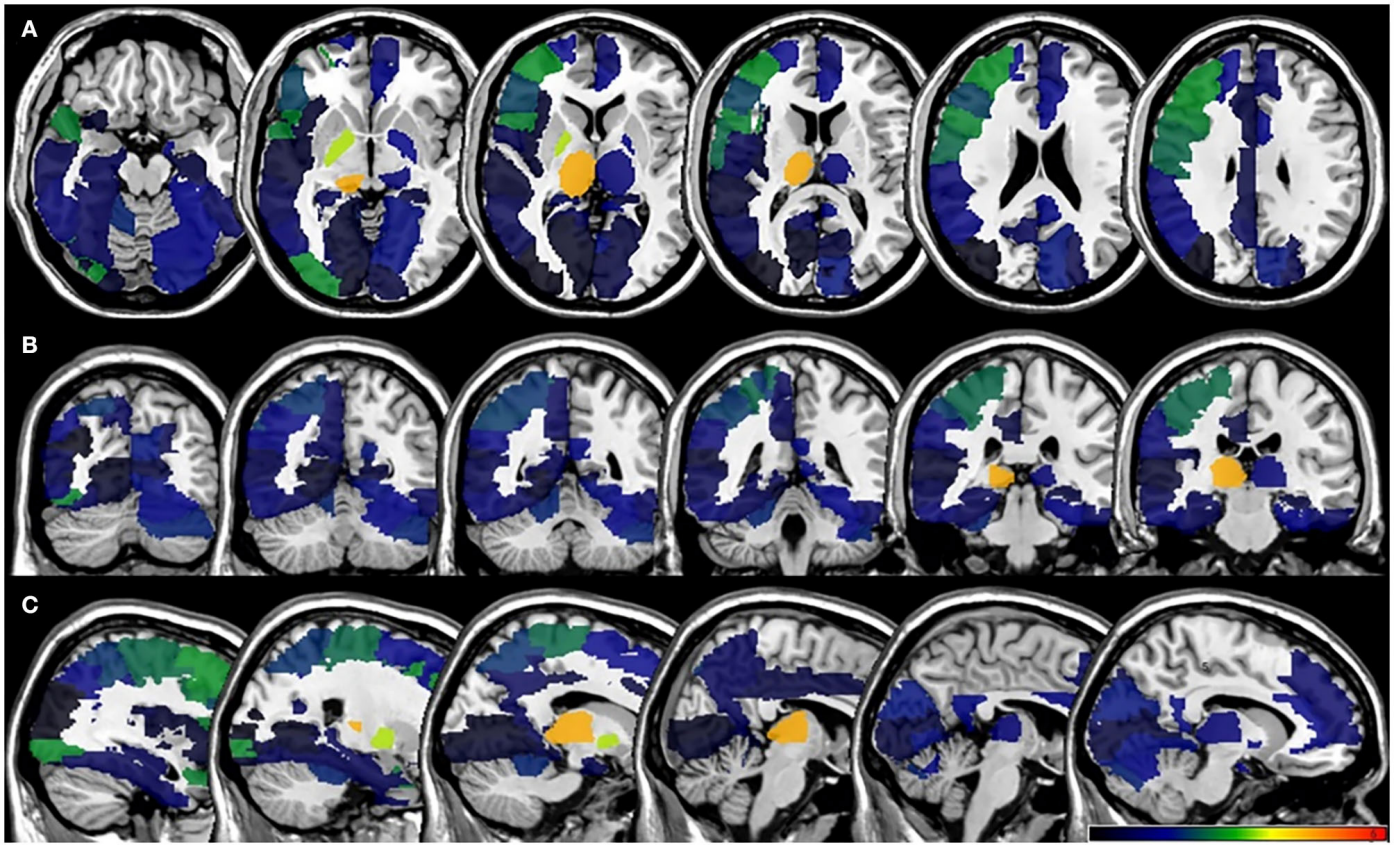
VLSM results for poststroke fatigue at 6-month follow-up. **(A)** axial plane, **(B)** coronal plane, and **(C)** sagital plane. VLSM, voxel-based lesion-symptom mapping.

In the VLSM analyses, we did not find significant lesion-behavioral relation between patients with and without PSF in the acute phase of the stroke (Figure 3). No significant cluster was found when FSS was treated as a continuous variable (Supplementary Figure 2). For the fatigue assessment at 6-month follow-up, VLSM analysis revealed that the voxel cluster in the right thalamus was significantly associated with PSF (Figure 4). In addition, the severity of PSF (FSS score) was also associated with the damage in the right thalamus (Supplementary Figure 3).

**Figure 5:**
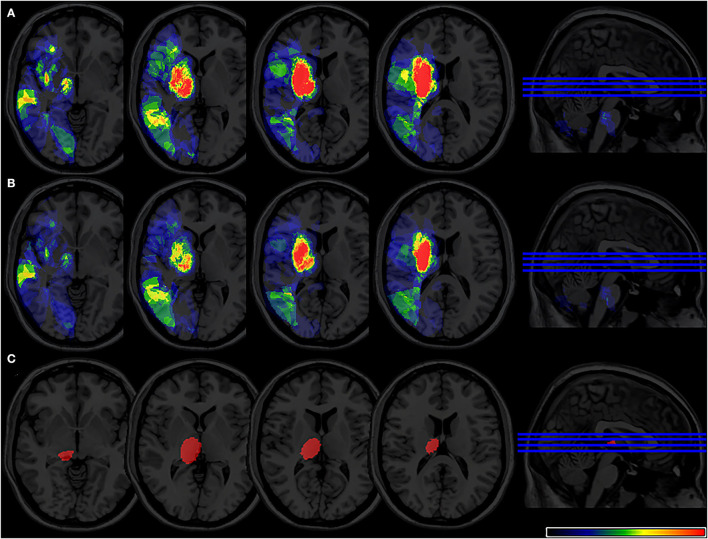
**(A)** Lesion probability map of 344 patients (17 were excluded due to bilateral lesions) after lesion left-right flipped in the acute phase. **(B)** Lesion probability map of 309 patients (15 were excluded due to bilateral lesions) after lesion left-right flipped at 6-month follow-up. **(C)** 2 VLSM results for poststroke fatigue at 6-month follow-up after flipped analysis. VLSM, voxel-based lesion-symptom mapping.

Participants in our study include right- and left-sided lesions, so we flipped left-sided lesions to right hemisphere along the midsagittal line and the same VLSM analysis was performed (Figure 5). In total, 15 patients were excluded due to bilateral lesions. Similarly, VLSM analysis revealed that thalamus lesions also associated with PSF at 6-month follow-up after stroke onset.

### Multivariate Analysis of the Correlation Between Lesion Location and PSF

**Figure 6:**
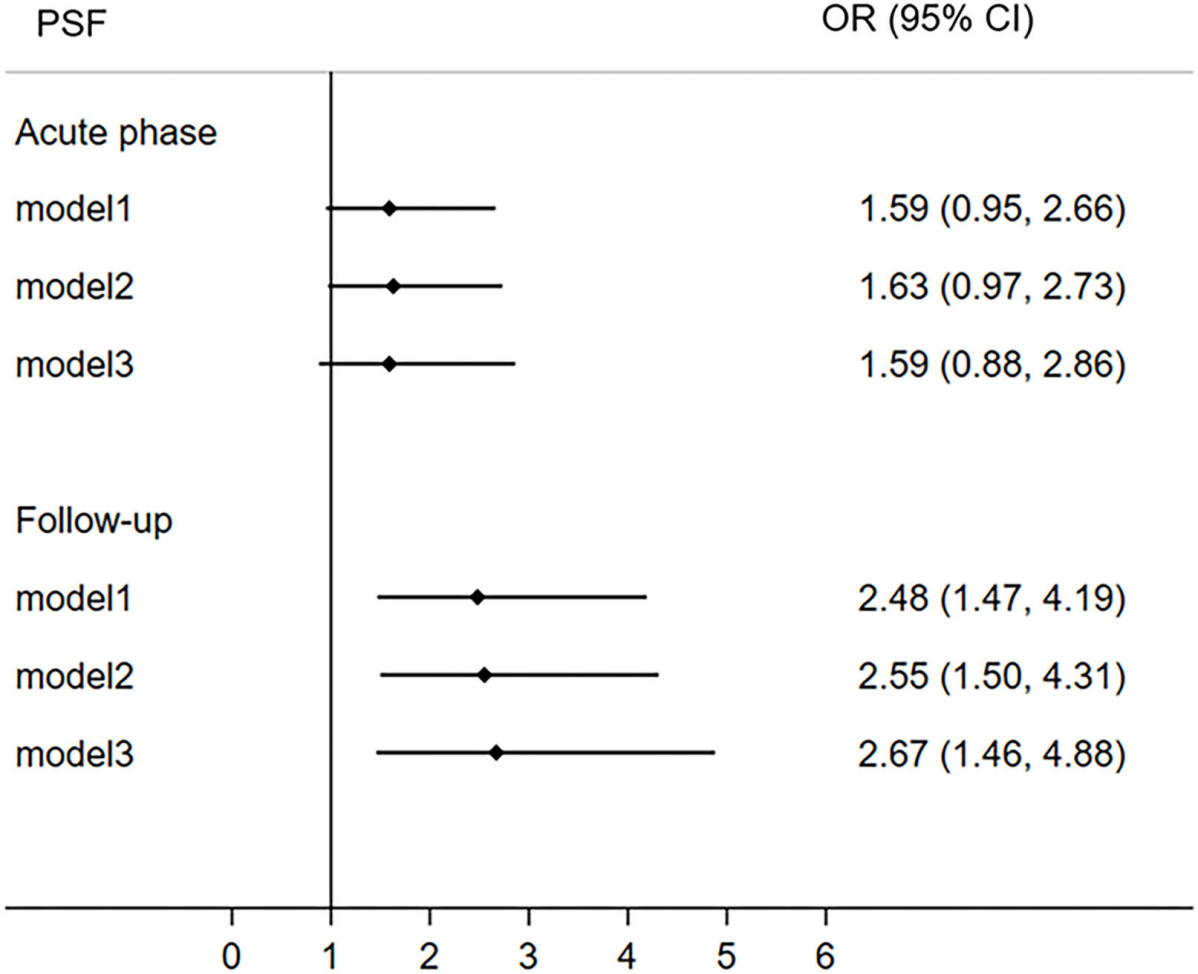
The correlation between right thalamus and post-stroke fatigue both in the acute phase and 6-month follow-up. The right thalamus comes from the result of VLSM analysis of poststroke fatigue at 6-month follow-up. CI, confidence interval; OR, odds ratio; PSF, post-stroke fatigue; VLSM, voxel-based lesion-symptom mapping.

The participants were divided into two group (VLSM+/VLSM–) based on the VLSM results at 6-month follow-up. More specifically, patients in VLSM+ group presented ischemic lesions in the right thalamus but VLSM– group without this infarct lesion in this area. The univariate analysis indicated that the hypertension, diabetes, lesion side, lesion volume, TOAST, NIHSS score, and 6-month PSF were significant difference between VLSM+ and VLSM– groups (Table 2). Multivariate logistic regression analyses revealed that right thalamus lesion was associated with 6-month fatigue (OR 2.67, 95% CI 1.46–4.88), but not with acute phase fatigue (OR 1.59, 95% CI 0.88–2.86; Figure 6).

**Table 2:** Baseline characteristics between VLSM+ and VLSM– groups.

	**VLSM– (*n* = 246)**	VLSM+^*^(*n* = 78)	***P*** **-value**
Age, mean (SD), y	59.8 (12.5)	57.1 (12.9)	0.091
BMI, mean (SD), kg/m^2^	24.6 (3.2)	25.4 (3.8)	0.064
Male, n (%)	159 (64.6)	46 (59)	0.366
Hypertension, *n* (%)	182 (74)	47 (60.3)	0.020
Diabetes, *n* (%)	97 (39.4)	21 (26.9)	0.045
Hyperlipidemia, *n* (%)	52 (21.1)	14 (17.9)	0.542
Smoking, *n* (%)	89 (36.6)	25 (32.5)	0.507
Drinking, *n* (%)	58 (23.9)	19 (24.1)	0.885
lesion volume, median (IQR), ml	3.0 (1.1–7.6)	2.3 (1.0–4.5)	0.017
Time to imaging, median (IQR), day	3 (2, 5)	3 (2, 5)	0.515
TOAST, *n* (%)			0.029
LAA	114 (46.3)	28 (35.9)	
SAD	53 (21.5)	12 (15.4)	
Others^a^	79 (32.1)	38 (48.7)	
NIHSS, median (IQR)	3 (1.7)	2 (0.4)	0.023
mRS, median (IQR)	1 (1.3)	1 (1.2)	0.146
HAMD, median (IQR)	4 (2.12)	4 (2.9)	0.916
HAMA, median (IQR)	4 (1–10.5)	5 (2–20.3)	0.017
LSNS, median (IQR)	28 (17–38)	30 (16.5–34.3)	0.706
PSF in the acute phase, *n* (%)	91 (37)	38 (48.7)	0.065
PSF at follow up, *n* (%)	75 (30.5)	41 (52.6)	<0.001
FSS in the acute phase, median (IQR)	3.1 (2.3–4.4)	3.7 (2.6–4.7)	0.073
FSS at follow up, median (IQR)	2.9 (2.0–4.2)	4.0 (2.8–4.5)	0.023

*BMI, body mass index; HAMA, Hamilton anxiety scale; HAMD, Hamilton depression scale; IQR, interquartile range; LAA, large artery atherosclerosis; LSNS, Lubben social scale; mRS, modified Rankin scale; NIHSS, NIH stroke scale; PSF, poststroke fatigue; SAD, small artery occlusion; SD, standard deviation; TOAST, trial of org 10172 in acute stroke treatment*.

a *Others, cardioembolism, stroke of other determined cause, and stroke of undetermined cause*.

* *VLSM+ refer to the result of the VLSM analysis at 6-month follow-up, which means that the infarct was located in the right thalamus*.

## Discussion

In this study, we used VLSM analysis to explore the correlation between focal infarct lesions and the fatigue symptom during the acute phase of ischemic stroke and at 6-month follow-up. We found that the lesions in the right thalamus might increase the risk of fatigue symptoms at 6-month poststroke while lesion locations were not associated with fatigue in the acute phase. Meanwhile, we also found the severity of fatigue was correlated with the lesions in right thalamus at 6-month follow-up.

A recent fMRI study indicated that PSF was associated with the posterior hypoactivity and prefrontal hyperactivity reflecting that dysfunction within fronto-striatal-thalamic and frontal-occipital networks may be involved in the occurrence of fatigue, which was partially consistent with our findings (Cotter et al., 2021). In addition, the prevalence of PSF in this study was 39.8% in the acute phase of stroke and 35.8% at the 6-month follow-up. This prevalence change was in line with the previously published articles (Christensen et al., 2008; Wang et al., 2018). Furthermore, we found that lesion laterality, anxiety, and depression symptoms were significantly different between fatigue and non-fatigue groups at 6-month follow-up after the stroke onset. The right hemisphere is primarily responsible for emotional regulation and has also been reportedly related to PSF in the previous literature (Mutai et al., 2017); Moreover, psychological factors play an important role in the persistent fatigue, which is consistent with the previous study (Kirchberger et al., 2022) and Chalder's conceptual model of PSF (Wu et al., 2015).

In the last decade, the relationship between PSF and lesion location did not reach a consensus. Some studies suggested that lesion location might be an important determinant of PSF, while others did not (Mead et al., 2011; Thilarajah et al., 2018). This could be explained by the relatively low sensitivity of method applied in these studies, because they all distinguished the lesion location by the visual evaluation and used clinical statistics to determine the correlation between the lesion location and PSF. The Winward's study indicated that fatigue symptom is more frequent in patients with minor stroke than transient ischaemic attack, suggesting that lesion itself might play an important role in the development of PSF (Winward et al., 2009). However, a structural brain disconnectivity mapping study indicated that the poststroke fatigue was not associate with lesion and structural brain disconnectome, which may contradict with our results. This consistency in results could be attributed to difference in study design, race difference, and small sample size (Ulrichsen et al., 2021).

In this study, VLSM analysis identified that the occurrence and severity of PSF were associated with the damage in the right thalamus at 6-month follow-up. One possible explanation is that the thalamus plays an important role in signal transmission in the brain, especially thalamocortical neuronal pathways could link to various emotional, motivational, and cognitive functions (Wilkinson et al., 2017). In addition, patients with thalamus infarcts were more likely to be fatigue possibly because of the disturbance of the limbic–motor integration networks (Tang et al., 2010). The thalamus as an important part of parallel circuits that connect the basal ganglia and frontal cortex, its damage makes sensorimotor, limbic, and motor information cannot be transformed into efficient thought and action (Bolam et al., 2000). The disconnection of motivation and movement leads to reluctance to activity and formed a feeling of fatigue (Chaudhuri and Behan, 2000).

Another possible explanation is that previous study indicated that the thalamus damage associated with lower serotonin transporter bindings, which might directly or indirectly interfere with the brain serotonergic pathways (Pavese et al., 2010). The damage of this signaling pathway has been reported to be related to the development of fatigue in patients with stroke (Andersen et al., 1994; Kim, 2002; Kim et al., 2012).

In contrast with the fatigue in the 6-month follow-up, we did not find a clear association between specific lesion location and PSF in the acute phase of stroke. Although previous studies have reported similar findings (Kutlubaev et al., 2012), the underlying mechanisms remain unclear. It is speculated that patients with stroke in acute phase with greater disability and their mental stress state may amplify perceptions of fatigue and further mask the effect of lesion location on PSF.

This is a relatively large sample, prospective, and based on domain-specific analyses to explore the relationship between stroke lesions and PSF during the acute phase and at 6-month follow-up. However, there are several limitations that need to be addressed. First, this study is observational and therefore cannot infer causality directly; second, the area deemed to have sufficient overlap for the analysis is too small (Figure 1). The lesions analyzed in our study were located mainly in the bilateral basal ganglia region, some potentially emotion related lesions such as dorsolateral prefrontal cortex was not completely included in the VLSM analysis. Third, the VLSM analysis was limited to AAL template-based brain regions, which could not detect more focal lesion area within the thalamus. Forth, the imaging data was collected only once during the acute phase of the stroke, so we did not analyze the impact of dynamic changes in infarct lesions on PSF. Fifth, patients with serious cognitive and communication deficits were excluded from our study, which limits the generalization of our findings. Sixth, patients with acute ischemic stroke within 14 days were admitted to this study, the change of DWI signal may affect the evaluation of infarct size.

In conclusion, our study identified a significant association between lesion location in right thalamus and fatigue symptoms 6 months' poststroke. Brain activity is a complex process, so the role of thalamus in the brain circuit should be proved by further interventional studies or the functional MRI analysis.

## Data Availability Statement

The original contributions presented in the study are included in the article/Supplementary Material, further inquiries can be directed to the corresponding author/s.

## Ethics Statement

Written informed consent was obtained from the individual(s) for the publication of any potentially identifiable images or data included in this article.

## Author Contributions

JW, MG, and LX contributed to the concept and design of the study. JW conducted the data analysis and wrote the first draft of the manuscript. SJ and DY conducted the data analysis. PW, WS, and XL contributed to the study design, interpretation of results, and critical revision of the manuscript. All authors contributed to the article and approved the submitted version.

## Funding

This work was supported by the Natural Science Foundation of Anhui Province (2108085MH271) and Key Research and Development Plan Projects of Anhui Province (202104j07020049).

## Conflict of Interest

The authors declare that the research was conducted in the absence of any commercial or financial relationships that could be construed as a potential conflict of interest.

## Publisher's Note

All claims expressed in this article are solely those of the authors and do not necessarily represent those of their affiliated organizations, or those of the publisher, the editors and the reviewers. Any product that may be evaluated in this article, or claim that may be made by its manufacturer, is not guaranteed or endorsed by the publisher.
