# Supplementary material for: Association of Lesion Location and Fatigue Symptoms After Ischemic Stroke: A VLSM Study
Source: Front Aging Neurosci. 2022 Jun 29;14:902604. doi: 10.3389/fnagi.2022.902604 (PMC9277067; doi:10.3389/fnagi.2022.902604)
Supplement: Supplementary file 1 [file Data_Sheet_1.docx]

Table I The baseline characteristics between included and excluded patients

|  | included | excluded | p value |
| --- | --- | --- | --- |
|  | (n=324) | (n=309) |  |
| Age, mean (SD), y | 59.2 (12.6) | 60.3 (12.6) | 0.259 |
| BMI, mean (SD), kg/m^2^ | 24.7 (3.4) | 24.8 (3.4) | 0.792 |
| Male, n (%) | 205 (63.3) | 187 (60.5) | 0.476 |
| Hypertension, n (%) | 229 (70.7) | 213 (68.9) | 0.632 |
| Diabetes, n (%) | 118 (36.4) | 103 (33.3) | 0.415 |
| Hyperlipidemia, n (%) | 66 (20.4) | 58(18.8) | 0.612 |
| Smoking, n (%) | 114 (35.6) | 102 (33.4) | 0.566 |
| Drinking, n (%) | 77 (24.1) | 80 (26.2) | 0.532 |
| TOAST, n (%) |  |  |  |
| LAA | 142 (43.8) | 151 (48.9) | 0.383 |
| SAD | 65 (20.1) | 61 (19.7) |  |
| Others ^a^ | 117 (36.1) | 97 (31.4) |  |
| NIHSS, median (IQR) | 3 (1-6) | 3 (2-8) | 0.056 |
| mRS, median (IQR) | 1 (1-3) | 1 (1-3) | 0.229 |

Abbreviations: BMI, body mass index; IQR, interquartile range; LAA, large artery atherosclerosis; mRS, modified Rankin Scale; NIHSS, NIH Stroke Scale; SAD, small artery occlusion; SD, standard deviation; TOAST, Trial of Org 10172 in Acute Stroke Treatment.

^a^ Others, cardioembolism, stroke of other determined cause, and stroke of undetermined cause.


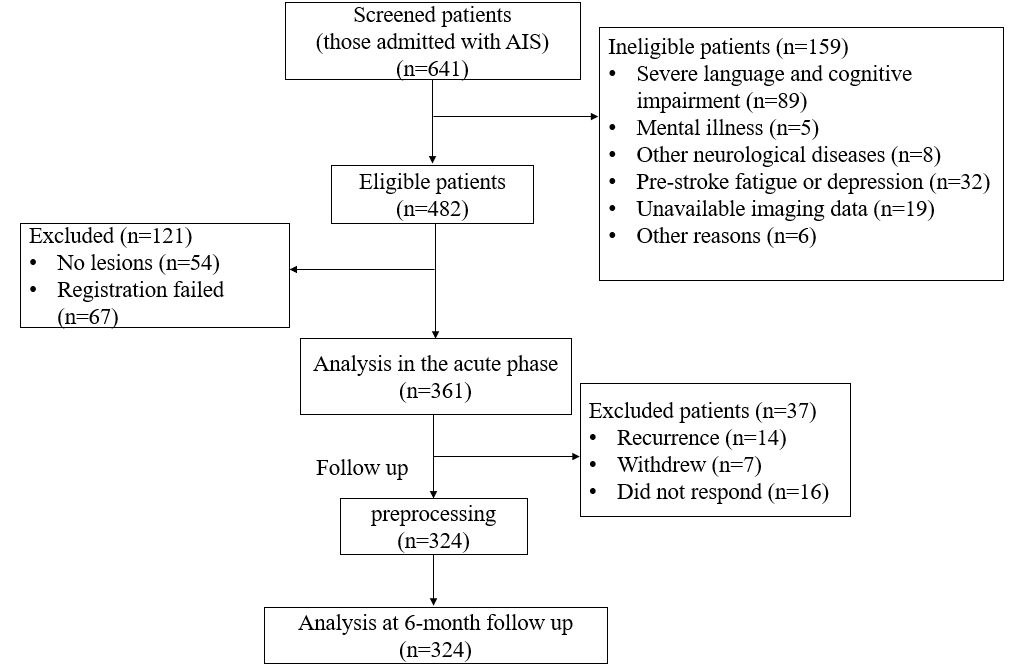


**Figure I** Flowchart of patient inclusion. Abbreviations: AIS, acute ischemic stroke.


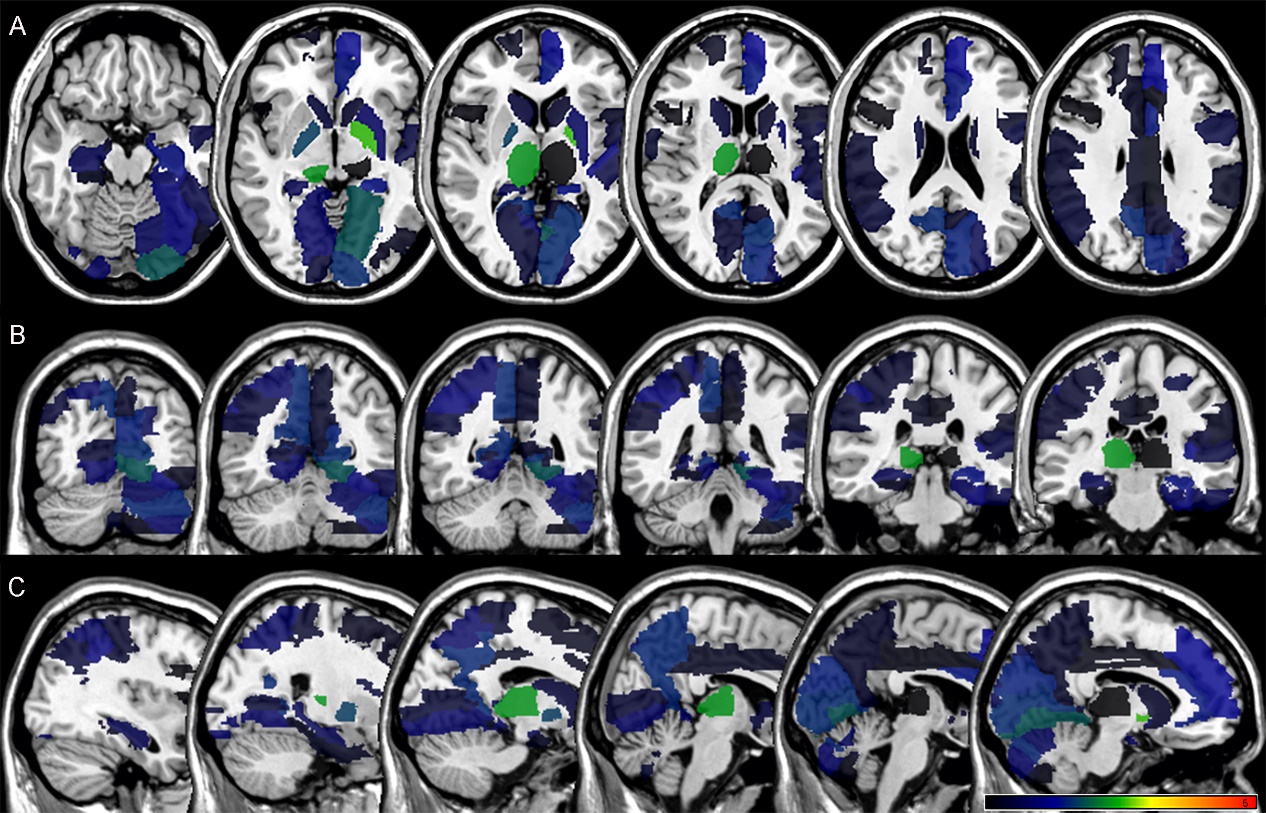


**Figure II** VLSM results for fatigue stroke severity in the stroke acute phase. Abbreviations: VLSM, voxel-based lesion-symptom mapping.


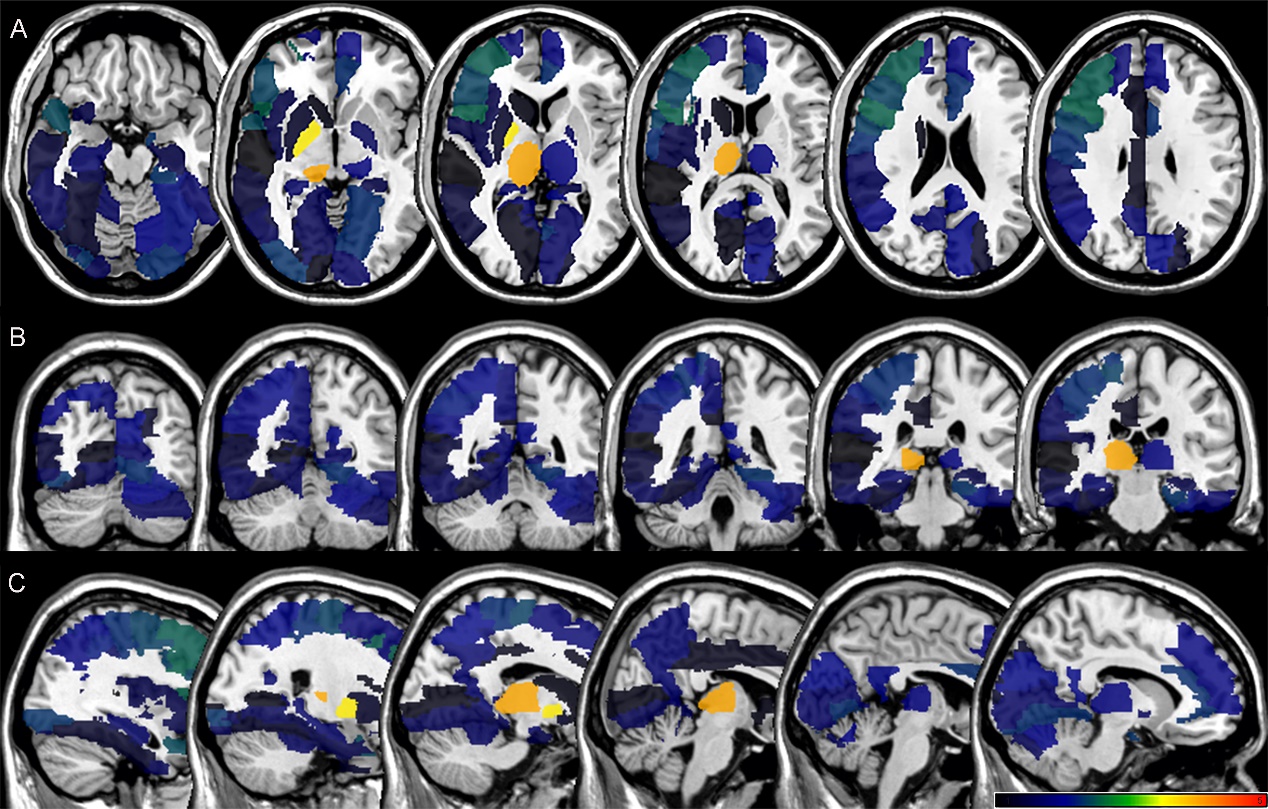


**Figure III** VLSM results for fatigue stroke severity at 6-month follow-up. Abbreviations: VLSM, voxel-based lesion-symptom mapping.
